# Two Different Points of View through Artificial Intelligence and Vector Autoregressive Models for Ex Post and Ex Ante Forecasting

**DOI:** 10.1155/2015/409361

**Published:** 2015-10-13

**Authors:** Alev Dilek Aydin, Seyma Caliskan Cavdar

**Affiliations:** Faculty of Business, Haliç University, 34200 Istanbul, Turkey

## Abstract

The ANN method has been applied by means of multilayered feedforward neural networks (MLFNs) by using different macroeconomic variables such as the exchange rate of USD/TRY, gold prices, and the Borsa Istanbul (BIST) 100 index based on monthly data over the period of January 2000 and September 2014 for Turkey. Vector autoregressive (VAR) method has also been applied with the same variables for the same period of time. In this study, different from other studies conducted up to the present, ENCOG machine learning framework has been used along with JAVA programming language in order to constitute the ANN. The training of network has been done by resilient propagation method. The ex post and ex ante estimates obtained by the ANN method have been compared with the results obtained by the econometric forecasting method of VAR. Strikingly, our findings based on the ANN method reveal that there is a possibility of financial distress or a financial crisis in Turkey starting from October 2017. The results which were obtained with the method of VAR also support the results of ANN method. Additionally, our results indicate that the ANN approach has more superior prediction performance than the VAR method.

## 1. Introduction

From the Great Depression in 1929 until the recent financial crisis of 2008, the world economy has experienced several financial crises that had grievous impacts on both industrialized and developing countries such as the Tequila crisis in 1994 in Mexico, the 1984 savings and loans (S&L) crisis in the US [1], the Asian financial crisis in 1997 and 1998, the Russian financial crisis in 1998, the financial crisis in Brazil in 1999, and the financial crises in Turkey and in Argentina in 2001. All of these mentioned crises have some common features, which constituted the appropriate climate for those crises to occur, such as financial liberalization, weak financial and banking systems that are open to fraud, unsustainable macroeconomic policies such as high trade deficits, undisciplined budget policies and uncontrollable capital movements, and global economic conditions and political instability.

As Berg and Pattilo [2] point out, the integration of financial markets and the globalization have posed new challenges and created new problems for policymakers. Although the speed with which money can be transferred from one country to another has increased incredibly thanks to advanced technology in global communications, financial markets are generally late at seeing a crisis approaching. Researchers are aware that developing a single economic model for all types of crises is impossible. However, constructing an early warning system based on different variables can be useful in predicting the degree of crisis for different countries so that which countries are more fragile in a period of financial distress can be determined. Thus, timing of a crisis can be identified and policymakers and analysts can focus their attention and concentrate their efforts on the policy adjustments before the crisis deepens.

With the emergence of the 2008 global financial crisis, researchers accelerated their empirical works on prediction of financial crises and developing early warning systems. Due to the systemic risk and the contagious effect of the recent crisis, economists and policymakers realized how financial shocks can cause devastating consequences on the real economy. Small investors suffered a significant decline in the value of their stocks, which had serial negative impacts on such economic variables as household consumption spending, business investment spending, total demand, and employment rate [3]. In this context, one of the most important negative impacts of the recent crisis has been the substantial increase in the rate of unemployment. Although the severity of this problem varies significantly from one country to another, young people and women have been badly hit all around the world.

If the crisis had been predicted, economists and policymakers would have provided an opportunity to reduce the negative impacts of the crisis or even totally avoid them [4]. However, policymakers have never been successful in predicting financial crises because they have never been able to measure financial instability properly. To reduce the possibility of another financial crisis in the future, it is certainly necessary to learn from past experiences by analyzing the causes that led to the crisis. The recent global crisis has undoubtedly increased efforts on the creation of early warning models to predict and to reduce severity of future crises.

The major purpose of this study is to predict stock market crash and financial distress before financial crisis by using different macroeconomic variables such as the exchange rate of USD/TRY (USD), gold prices (GOLDPRICE), and the Borsa Istanbul 100 index (BIST). Our specific objectives in this study can be listed as follows:

1. To identify (if any) the causal relationship between macroeconomic variables such as gold prices, the exchange rate of USD/TRY, and the BIST 100 index.
2. To predict a potential stock market crash or a financial crisis by using different variables in the future in order to minimize their costs to the economy.

This paper provides a highly expandable approach to neural networks and examines the connection between classical econometric techniques. An extensive review of the literature indicates that ANNs are generally more suitable than linear models for forecasting macroeconomics and financial variables. Although this method has several weak aspects, ANNs exhibit excellent results in many fields. In contrast to many researchers in the field, who tend to adopt an all-or-nothing approach to this issue, we argue that neural networks should be considered as the most powerful processor as compared to classical econometric methods. The full potential of neural networks can be exploited by using them in conjunction with linear regression models. Hence, as Gonzalez [5] states, neural networks should be viewed as an additional tool to be included in the toolbox of macroeconomic forecasters.

This paper is composed of four sections including introduction. The second section examines the main empirical literature which employs either the ANN method or the VAR method to test the relationships between different variables to predict financial distress or stock market crashes before the financial crises. The third section explains the empirical methodology used in the study and presents a detailed explanation of the empirical findings with their interpretations. The fourth section is the conclusion part.

## 2. Literature Review

In the last few decades, ANNs have been frequently used to solve complicated problems and to test the relationship between different variables. Empirical researches clearly indicate that they can be used as efficient tools to analyze critical issues in business decisions. In this context, ANNs have many advantages which may help to solve problems in finance as well as in other fields with poorly defined models. As Tu and Oztemel [6, 7] set forth, main advantages of the ANN include their ability to detect complex nonlinear relationships between independent and dependent variables, their ability to execute machine learning, their self-organization and self-learning skills, their error tolerance capabilities, and their ability to learn by using examples and to work with inadequate information. Neural networks have been useful to predict volatile financial variables that are quite difficult to guess with classical statistical and econometric methods, such as exchange rate and stock market [8, 9]. Neural networks have also been successfully applied to macroeconomic variables such as economic growth [10] and industrial production [11].

Many researchers prefer to utilize ANN to conduct their studies in different fields. However, only a limited number of researchers prefer to use ANN in their empirical researches to identify financial distress, to develop early warning systems, or to predict financial crisis. In one of these studies, Nag and Mitra [12] utilized the ANNs to develop an early warning system to detect exchange rate crisis for Indonesia, Malaysia, and Thailand in the time period between 1980 and 1998. They employed sixteen different variables for their research and compared their results with the results obtained by the signal approach. They conclude that the ANN model has a superior performance of prediction as compared to the signal approach.

Franck and Schmied [13] used the ANN model in the prediction of speculative attacks in Russia and Brazil in 1998 and 1999. They observed that trying to determine the nonlinear relationships by using linear models weakens the possibility of the prediction of financial crises. However, they asserted that they obtained a more accurate performance as compared to the logit models in the prediction of speculative attacks by using ANNs in their study.

Ozkan-Gunay and Ozkan [14] used ANN as an inductive algorithm to explain previous bank failures in the Turkish banking sector as a significant example of an emerging financial market. They found that most of these banking failures could actually be predicted long before with the use of ANN method. They also claimed that detecting early warning signals of potential failures as in the case of the Turkish banking sector would be possible. Most importantly, their study indicates that ANN can be used as a strategic method of reviewing financial variables in terms of predictive accuracy, adaptability, and robustness as an alternative early warning method that can be used with other most common alternative methods.

Fioramanti [15] tried to predict the debt crisis in less developed countries by using the data that belong to the period between 1980 and 2004 by utilizing the ANN model. He argues that the ANN model can provide more accurate results as compared to other early warning systems thanks to its high flexibility and its ability to analyze the nonlinear relationships. In his study, he employed yearly data of 46 developing countries that involved 34 independent variables. Fioramanti [15] also observed that the ANN model provided more accurate and successful results.

Ban and Mazibas [16] developed neural network models to classify failed and nonfailed banks prior to their failure. When they conduct their study, they used the financial and operational ratios of 36 selected banks operating in Turkey as an input to the two types of neural network structures which are Multilayer Perception (MLP) and Generalized Feed Forward Networks (GFNs). The researchers developed ten ANN models with a variety of hidden layers and perceptrons in each layer. They also compared alternative models in terms of classification accuracy and estimated their models by employing the data, which belong to the period between 1995 and 2000. Additionally, they conducted their out-of-sample forecast comparisons for the period between 1997 and 2000 for each year, before the bank failures. In addition to the ANN models, they used discriminant and logistic regression models to compare their classification performances. Their results revealed that the ANN outperformed other models.

Several other researchers choose to employ VAR model to determine the relationships between different variables which might be used to predict stock market crashes or financial crises. As Shinkai and Kohsaka [17] state, endogeneity problem can be avoided by using the VAR method. With the VAR method, by treating all variables as endogenous, we can obtain exogenous financial shocks and thus avoid the endogeneity problem. VAR model is also one of the most successful and flexible models for the analysis of multivariate time series in order to model causal relations and joint dynamics among a variety of macroeconomic variables. Some major researchers, who have made significant contributions to the empirical literature concerning modeling time series with VAR, include Hamilton [18], Culbertson [19], Campbell and Mackinlay [20], Mills [21], and Tsay [22].

Dhakal et al. [23] are among the researchers, who prefer to use VAR model to test the relationships between stock prices and macroeconomic factors. Reside and Gochoco-Bautista [24] and Fujii [25] studied the relationship between exchange rates. Daly [26] and Yang et al. [27] identified the relationships among the stock markets. Wu [28] and Phylaktis and Ravazzolo [29] tested the interaction between the exchange rate and the stock market. Chen [30] identified the relationship between money supply (M1) and stock price by utilizing a VAR model for Granger causality test. All these studies have a common point that they use similar methodology to analyze the relationship among the macroeconomic variables. They utilize either VAR or Vector Error Correction (VEC) model, to model the variables to identify short-term or long-term relationship among these variables.

Sengonul et al. [31] utilized a VAR model for the period between 1990 and 2005 to analyze the interactions between the short-term capital inflow and real variables such as Gross Domestic Product (GDP) growth and investment and financial variables such as interest rates, exchange rates, and inflation. Researchers found that short-term capital inflow leads to a negative trade balance by appreciating domestic currency and increases interest rates in the long term. Consequently, volatile characteristics of short-term capital inflows have increased instability in the economy and resulted in a sensitive economic environment that is open to potential crises.

Bayraci et al. [32] constructed a VAR model to predict the interaction between BIST 100 index, the exchange rate of TRY/USD, and short-run interest rates in the time period between 2006 and 2010. Their findings indicate that VAR model is a satisfactory model for interest rates and exchange rates. However, it did not give satisfactory results for the stock market dynamics.

Koc and Akgul [33] aimed to present structural regime change periods of Turkish economy between 1990 and 2007. In their research, they utilized Markov Switching Autoregressive (MS-VAR) models to determine these regime structures. They conducted their analysis by using various macroeconomic variables such as current account, GDP growth rate, ratio of import coverage by export, and interest rate series for the period between 1992 and 2007. By determining three different regimes in this period, their study contributed positively to reduce uncertainty and to assist in decision-making in money and financial markets.

Zhou and Zhang [34], in their study, tried to predict stock market crash before financial crises with the aim of mitigating potential losses. They decided to select Granger causality test and VAR model to be used in their research to test the time lag. The sample period for their study is between 1987 and 2013. They employed the macroeconomic variables of exchange rate, Hong Kong Interbank Offer Rate (HIBOR), money in circulation, and T-Bill rate to conduct their studies. By using VAR Lag Order Selection Criteria, they found that they can use one or two months forward HIBOR to predict stock market crash in Hong Kong index in the future.

## 3. Methodology and Findings

### 3.1. Vector Autoregressive (VAR) Models

VAR model is an easy to use model for the analysis of multivariate time series. It has been proven to be especially useful for describing the dynamic behavior of economic and financial time series and for forecasting. With VAR models, it is possible to approximate the actual process by arbitrarily choosing lagged variables. Thereby, one can form economic variables into a time series model without an explicit theoretical idea of dynamic relations. The easiest multivariate time series model is the bivariate VAR model with two dependent variables, *y*_1,*t*_ and *y*_2,*t*_, where *t* = 1,…, *T*. The development of the series should be explained by the common past of these variables. That means the explanatory variables in the simplest model are *y*_1,*t*−1_ and *y*_2,*t*−1_. The VAR(1) model with lagged values for every variable is determined by

(1) y1,t=α11y1,t−1+α12y2,t−1+ε1,ty2,t=α21y1,t−1+α22y2,t−1+ε2,t.

The error terms *ε*_*it*_ ~ i.i.d(0, *σ*_*εi*_^2^) are assumed to be white noise processes, which may be contemporaneously correlated, but are uncorrelated with any past or future disturbances [35].

VAR analysis assumes that all the stochastic processes in the autoregressive system are variance-covariance stationary. If this condition is not met, little confidence can be placed in the VAR regression results. Since most if not all of the data here are highly autocorrelated and tend to drift over time, some form of detrending is obviously required before the models can be estimated. Our choice was influenced in part by the recent work of Dickey and Fuller [36]. The Augmented Dickey Fuller (ADF) test results from VAR model are shown in Table 1. We decided to use monthly data of three variables, which are the exchange rate of USD/TRY (USD), BIST 100 index (BIST), and gold prices (GP) over the time period between 2010 and 2014 to build our model. An important preliminary step in model building and impulse response analysis is the selection of the VAR lag order. In this study, we use some commonly used lag-order selection criteria to choose the lag order, such as the Akaike Information Criteria (AIC), the Hannan-Quinn Criterion (HQC), and the Schwarz Information Criterion (SBC).

However, some situations exist when SBC has the highest rate of picking the right lag order compared to HCQ and there are also situations when HCQ outperforms SBC. Nevertheless, compared to Monte Carlo simulation studies, the true model is not known in empirical research. Thus, when these two information criteria are selected according to the two different lag orders, it is quite difficult to determine which criterion one should rely on. Some researchers suggest linking these two criteria to obtain the performance of the above information criterion based on Monte Carlo simulation experiments for choosing the optimal lag order in stable and unstable VAR models [37]:

(2) HJC=ln⁡det⁡Ω^j+jn2ln⁡T+2n2ln⁡ln⁡T2T,j=0,…,K,

where ${\hat{\Omega }}_{j}$ is the maximum likelihood estimate of the variance-covariance matrix *Ω* when the lag order used in estimation is *j*. *T* shows the sample size. An alternative information criterion suggested by Hatemi-J [37] is

(3) $$\begin{array}{c} HQC=\ln\left(\;\det{\hat{\Omega }}_{j}\;\right)+j\frac{2n^{2}\ln\left(\ln T\right)}{T},\;j=0,\ldots ,K. \end{array}$$

To choose the optimal lag order in the VAR model, Hannan and Quinn [38] suggested the following information criterion (SBC):

(4) $$\begin{array}{c} SBC=\ln\left(\;\det{\hat{\Omega }}_{j}\;\right)+j\frac{n^{2}\ln T}{T},\;j=0,\ldots ,K. \end{array}$$

Table 1 presents the frequency distributions for the information criteria presented by (2). These results indicate that the rate of choosing the right lag order is 97.5% for stable VAR models and 95.3% for unstable VAR models. 180 observations were used in the simulations. Our findings showed that the percentage point of picking the right lag order even increased (over 90%) without taking account stability or instability assumption of the VAR into consideration. The mean lag was also calculated, which was 1.98 for stable VAR models and 2.01 for unstable VAR models.

Table 2 shows the results of the unit root tests with the 5% Mackinnon critical values, which indicates that all the variables are nonstationary; specifically, the variables are *I*(1).

**Figure 1:**
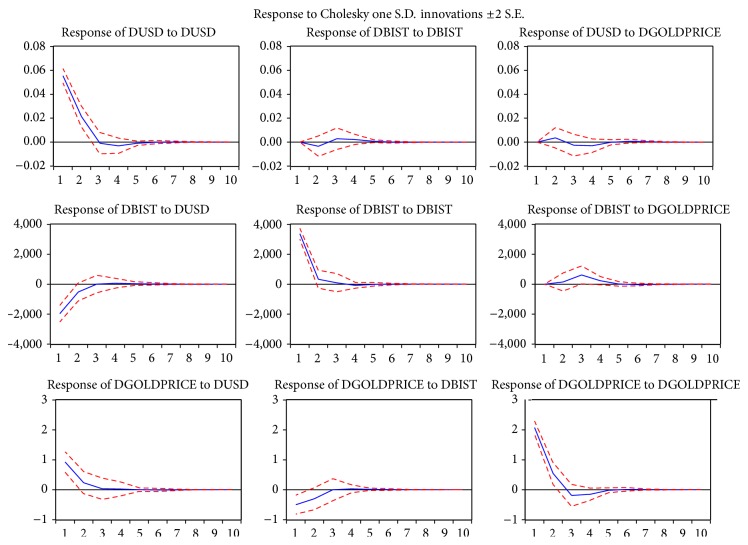
The results of impulse response analysis with VAR model.

The impulse response analysis quantifies the reaction of every single variable on an exogenous shock to our model. The impulse response function of VAR is to analyze dynamic effects of the system when the model receives the impulse. Impulse response analysis based on vector autoregressions (VARs) has an important place in modern empirical macroeconomics (for reviews of this literature, see Christiano et al. [39] and Pesaran and Smith [40]). As many researchers, we also study impulse responses in structural VAR models based on identifying assumptions about the short-run and long-run responses of the economy to individual structural shocks (e.g., Shapiro and Watson [41]; Blanchard and Quah [42]). In our VAR model, we have three variables, which are the exchange rate of USD/TRY (USD), BIST 100 index (BIST), and gold prices (GOLDPRICE). From Figure 1, we can see that when the impulse is BIST, every response of GOLDPRICE is positive at each time of the response period. Almost half responses of USD are positive and the value fluctuates around the line zero. On the other hand, every response of BIST is mostly negative. In other words, a standard deviation shock to BIST increases GOLDPRICE. Gold prices reach a maximum about 3 months after the initial BIST shock in the economy.

The right column shows the impulse responses of other variables to the gold prices. When the impulse is GOLDPRICE, the response of USD is firstly positive. There is the highest positive effect in the second month, and the lowest negative effect is in the sixth month. The response of BIST is a smooth fluctuation, and more varieties in terms of fluctuations exist starting from the second month. In other words, a standard deviation shock to the GOLDPRICE causes gold prices suddenly to create a negative effect and this effect remains until the third month. After the turning point, the value fluctuates around the line zero. One standard deviation shock to USD causes USD to peak about 1-2 months. Then, it begins to decrease eventually leading to a substantial drop in USD after about 5-6 months. A standard deviation shock to GOLDPRICE increases the USD, and the effect becomes statistically significant 2 months after the shock. USD decreases to its previous value after about 6 months.

Variance decomposition analysis tries to show how a change of a variable is affected from other variables. In this study, we will explain the variance of the variable until the 10th period in variance decomposition here. According to the results of the variance decomposition of GOLDPRICE in Table 3, by excluding the impact of output itself, we found that the most important variable is BIST 100 index (BIST) (48.41%). The table reveals that the second most important variable is the exchange rate of USD/TRY (USD) (0.32%). The most important variable for BIST variable becomes gold prices approximately after 10 periods (16.37%). The results of variance decomposition for USD show that the most important variable is gold prices. This is an interesting finding when we consider that the most affected variable from gold price is not actually the variable of USD. It can be interpreted as the USD has been less effected directly from GOLDPRICE in financial markets while gold price has been effected more from the potential fluctuations of USD.

### 3.2. Artificial Neural Networks (ANNs)

Over the last decade, a number of techniques have been developed, which allow estimation of general nonlinear models without specifying an exact functional form. Neural networks are one of these most popular techniques. White [43] made considerable work emphasizing the relationship between traditional classical statistics and the neural network theory.

**Figure 2:**
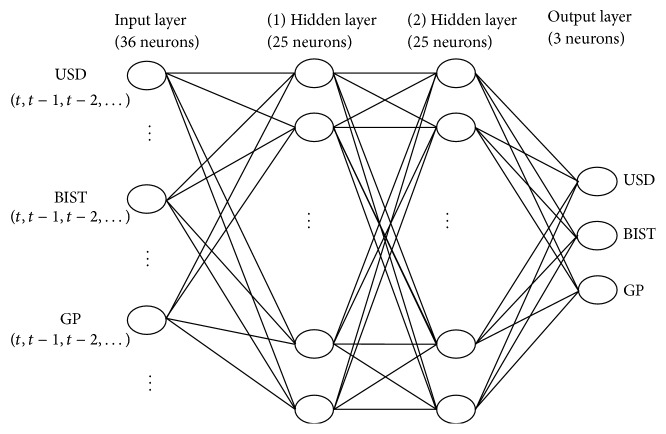
A brief multilayered feedforward neural network (MLFN) architecture of the proposed methodology for ANN.

In this paper, the data of 177 months between January 2000 and September 2014, obtained from the Electronic Data Distribution System of the Central Bank of the Republic of Turkey (CBRT) and the website of the World Bank, were used. In the empirical analysis part, three different variables, which are USD/TRY exchange rates (USD), gold prices (GP), Borsa Istanbul (BIST) 100 index, were used. In the prediction of the next 36 months of crisis indicator data in Turkey, we preferred to use feed-forward, backpropagation, multilayered ANN. It consists of an input layer, two hidden layers, and an output layer as shown in Figure 2. The independent variables represent the input vector and each value is provided to the network in one of these input layer nodes, where each node consequently represents an explanatory variable. The desired output is determined as the value of the output layer nodes. In other words, regardless of the total number of layers, each node is linked to all nodes in the previous and in the following layers. Obtained models have been investigating whether the data will show a decrease or increase in comparison with previous or latter month.

In this study, in terms of applied method, a feed-forward and multilayered ANN has been used which is composed of 3*∗*12 = 36 inputs, 3 outputs, and 2 hidden layers in which 25 neurons are included in each layer. In the layers of the network, the sigmoid activation function has been used. In addition to this, the resilient propagation learning algorithm has been used for the learning of the network. In this learning process, the error value of 0.1% has been determined as a target value, which provides a limit between the desired values. In the structure of a multilayered ANN, there are mainly three layers. The first layer is an input layer with artificial neural cells, which are connected to each other in different ways. The second one is the hidden (intermediate) layer. Finally, the third one is the output layer. In Figure 2, a three-layer network structure, artificial neurons, and interlayer relations have been demonstrated schematically. The input layer, which is composed of 36 (3*∗*12) data sets, is the first layer and it is responsible for transmitting incoming data in the ANN. It also serves to distribute the incoming data to the hidden (intermediate) layer. The neurons in the hidden layer, which process the information, do not have any links with the external environment. The output layer, which is composed of 3 data sets, processes the information coming from the intermediate layer. It also generates the outputs which are constituted for the input data presented by the input layer.

As seen in Figure 2, input layer consists of 36 neurons taking in 12 for each variable. We see that ANN is trained expeditiously with two hidden layers and 25 neurons for each hidden layer. Output layer also has 3 neurons. The proposed ANN structure here shows similarity to the study of Banik et al. [44] as they had two hidden layers in their research. In their study, they also had the similar nonlinear perspective when they formed their network in which they analyzed the high frequency time series data. Additionally, they utilized sigmoid function as the activation function similar to our research. In this study, ANN models have hidden layers and the ANN models, which do not have hidden layers, can be used in special cases where relationships between variables are linear. In other words, if what the ANN will find out is linear, the hidden layers do not exist in neural network models. Time series, generally, have high frequencies that the relationship between variables is supposed to be nonlinear. On the other hand, the number of hidden layers and the neurons in the hidden layers have been designated by the method of trial and error. More clearly, we determined the number of hidden layers, the neurons, and the best performing networks with the method of trial and error. Accordingly, we chose the most suitable network for our study. Furthermore, the normalization or denormalization of networks has been constituted by the built-in functions and normalization of numeric values which take part in ENCOG framework [45, 46].

Because of easiness in its utilization, time series, which take part in ENCOG framework, have been given preference in the Temporal Neural Dataset class of software that are constituted for the data sets. Additionally, we benefited from several strategies in order to assist coding in the framework such as “if certain number of iterations have been passed in the training of the network, restart to learning” or “if the error value does not progress a significant proportion, restart to learning.”

In this study, the normalization and the denormalization of data have been done with the help of built-in functions found in framework. While the network is being trained, the twelve-month lagged values of data were given as prologue and we demanded the forecasting of the 37th month. In the same manner, we tried to estimate results starting from the 37th month of 176 months that are in the data set. For each month to be predicted, ANN has been trained iteratively with its previous three-year data. For the 178th and the following months, again its previous three-year data has been given to the ANN as training set, and the missing months have been filled by the prediction values obtained in previous iterations. In other words, different networks have been used in our analysis in the calculation of each prediction value as compared to other studies which use a single network.

**Figure 3:**
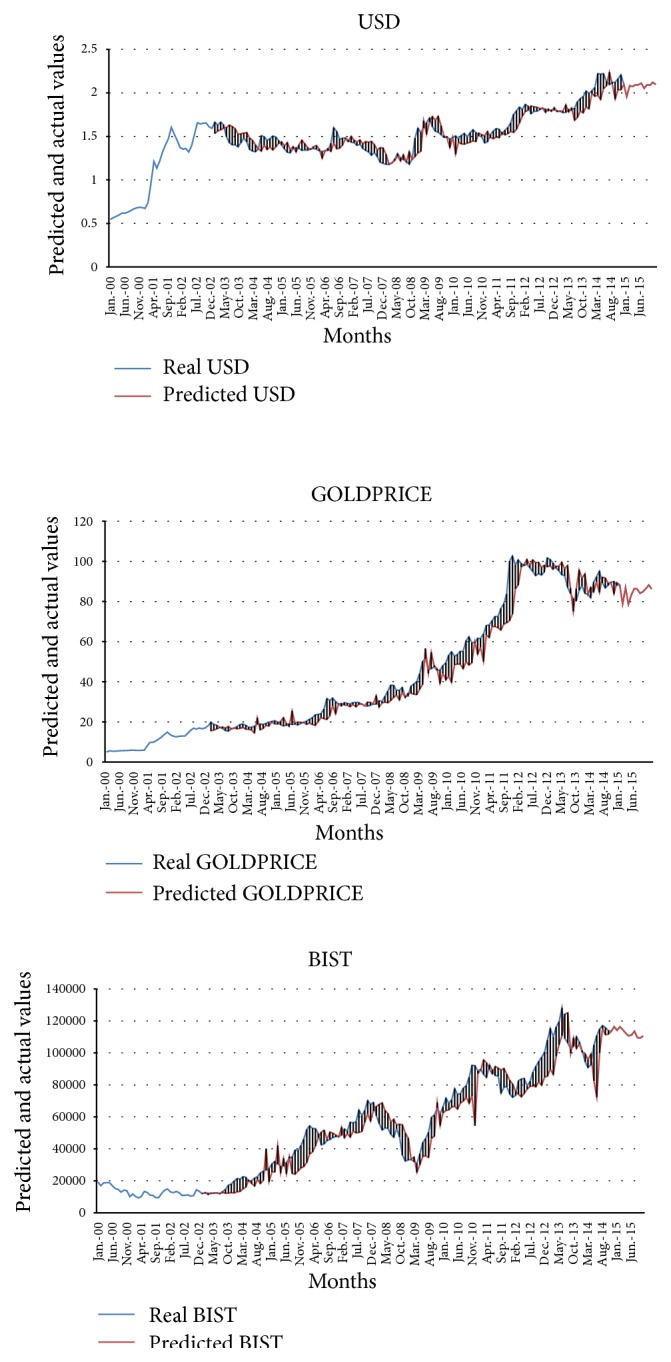
Comparison of real and predicted values with ANN.

Figure 3 plots the relationship between the real and predicted values for each month during 2000 and 2015. It can be seen from the figure that the real and the predicted values are very close to each other. We can see the impact of the recent global financial crisis on Turkish economy from the figure clearly by analyzing its effects on these three variables.

Turkish economy also experienced the major crises of 1994 and 2001 until 2008. During both crises, large amounts of capital entered and left Turkey, leading to instability in the exchange rate regimes. In the aftermath of the 1994 crisis, the Turkish economy contracted by 6% and the Turkish Lira was devalued by more than 50% against the US Dollar. The second crisis erupted in the midst of an exchange rate based stabilization program. In February of 2001, the fixed exchange rate system was collapsed and Turkey began to implement the floating exchange rate system. Instability in the financial markets and lack of confidence together with optimism in expectations all created speculative attacks. As a result of both of these crises, the trust to the Turkish Lira was shaken, the reserves of the Central Bank of Turkey decreased significantly, and inflation rate skyrocketed. The period of 2002 and 2007 was a stability period thanks to structural reforms applied in Turkish economy and the financial system and the positive course of events in the global economy. However, this stability period has come to an end with the 2008 financial crisis.

The 2008 financial crisis, the first global financial crisis of the world, has had a deep negative effect initially on developed countries and later on emerging countries. Turkey is one of these developing countries which have been affected negatively from this crisis. As it can be seen from Figure 3 that, as a result of the recent crisis, the exchange rate of USD/TRY has risen considerably since 2008. Gold prices have also climbed up sharply starting from 2008 till 2012. This trend indicates that gold prices have actually risen because investors have been in search of safety especially at the outset of the crisis. As the risk perceptions of investors decrease, gold prices also decline. BIST 100 index dropped substantially in 2008 but bounced back and entered into an increase trend. On the other hand, these trends were interrupted and the BIST 100 index dropped significantly in 2011 and 2014 as it happened in 2008.

**Figure 4:**
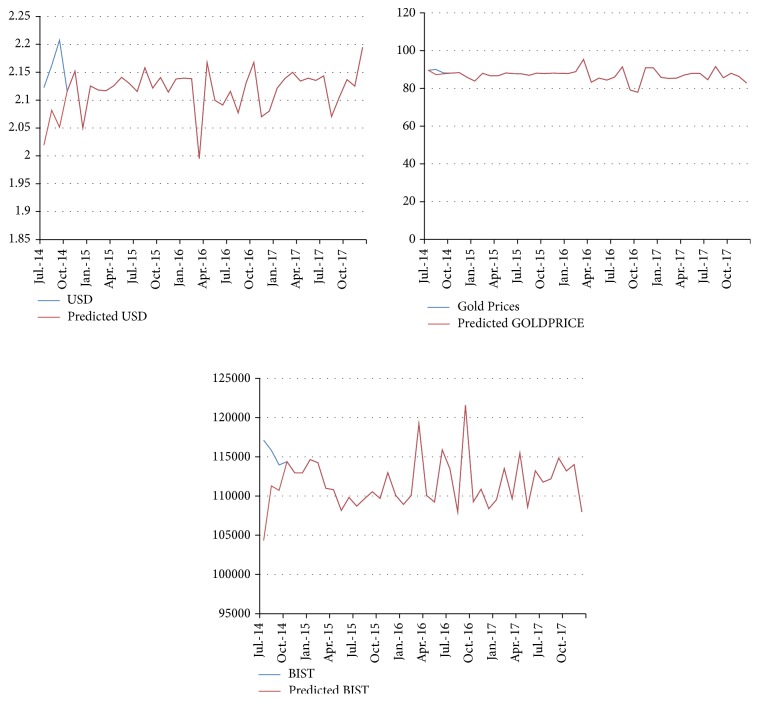
The future values predicted with ANN (2014–2017).

Figure 4 presents the future values of three macroeconomic variables obtained from the ANN. Except for gold prices, both the exchange rate of USD/TYR and the BIST 100 index fluctuate significantly between 2015 and 2017. Gold prices enter into a stable period except for April and October 2016. On the other hand, when the USD/TRY exchange rate drops sharply in April and October 2016, the BIST 100 index skyrockets, which indicates that these two variables are actually reversely correlated. When the data are interpreted in detail and previously experienced crisis in Turkish economy is taken into consideration, investors should note these dates carefully. In other words, there is a probability of a financial distress or a financial crisis starting from October 2017. Consequently, there may be significant amount of capital outflows and investors may demand higher risk premiums to compensate for their possible losses during this period.

**Figure 5:**
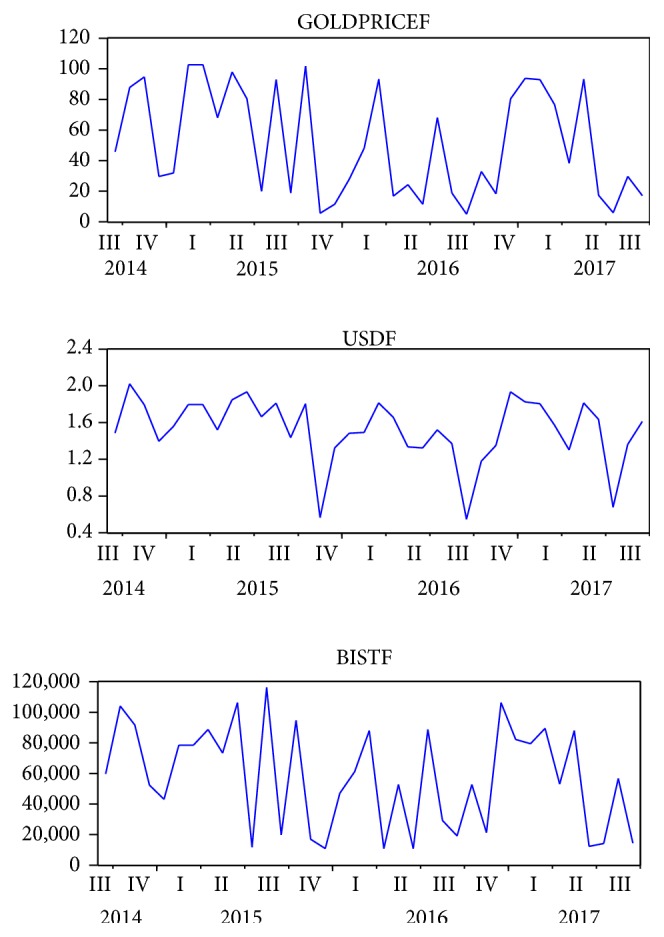
The future values predicted with VAR model.

Figure 5 demonstrates the future values predicted with VAR model. It, respectively, charts and compares the variables of gold prices (GOLDPRICE), the exchange rate of USD/TRY (USD), and the Borsa Istanbul 100 index (BIST) in Turkey. When it is examined in detail, the figure clearly demonstrates that all of these three variables will decrease sharply during the fourth quarter of the 2015 and the third quarters of 2016 and 2017. Although the most frequent fluctuations can be observed for the variable of BIST, the third quarter of 2016 and 2017 are expected to be more turbulent for the variables of GOLDPRICE and USD. Based on the real values, all of these fluctuations in given dates can be evaluated as the signs of potential crises for the Turkish economy. Interestingly, although there is a similarity between the two methods, these predictions do not correspond to the results that we obtained with the method of ANN.

## 4. Conclusion

The current financial crisis, the dynamism of financial markets, and the globalization process have accelerated the obsolescence of financial crisis prediction models and emphasized the need to reformulate these models. Neural networks arise as a powerful tool to enhance modeling flexibility and dynamism and to identify the most outstanding properties to predict financial crisis originated in some financial variables such as gold prices, stock exchanges, and exchange rates.

In this study, the causal relationship between macroeconomic variables such as gold prices, the exchange rate of USD/TRY, and the BIST 100 index has been investigated. Additionally by using these variables, we aimed to predict potential stock market crashes or financial crises in order to minimize their costs to the economy. We used two different methods of Artificial Neural Networks (ANNs) and the Vector Autoregressive (VAR) method to conduct this study. According to the findings of the ANN method, there is a possibility of significant fluctuations for the USD/TRY exchange rate and the BIST 100 index between 2015 and 2017 except for gold prices. Strikingly, our findings based on the ANN method reveal that there is a possibility of a financial crisis starting from October 2017.

Our results that we obtained with the VAR method is different from our results that we obtained with the ANN method. According to the results of VAR method, all of these three variables will fluctuate significantly between 2015 and 2017. However, very sharp fluctuations can be observed in the fourth quarter of 2015 and the third quarters of 2016 and 2017 for all of the variables. Based on our findings with the VAR method, we interpret that there is a possibility of a potential crisis in one of these dates.

Our study indicates that predictions of VAR method do not correspond to the ANN results. In this regard, it can be said that ANN method has a better estimation capability and we obtain more accurate results as compared to the VAR method. We come to this conclusion by analyzing the real values obtained in the past for both the methods of VAR and the ANN. Some of the major literatures, which support our perspective, include Nag and Mitra [12], Franck and Schmied [13], Fioramanti [15], and Ban and Mazibas [16].

The literature on building economic models and early warning systems for the prediction of financial crisis is promising but still new. The main difficulty in studying macroeconomic problems is how to model people's expectations when the economic environment is volatile mainly in developing countries. Small changes in expectations can often lead to large changes in people's behavior and, thus, in the behavior of macroeconomic variables such as GOLDPRICE, foreign exchange rates, and stock market exchanges.

As a suggestion for further research, researchers can obtain more accurate results and make more precise predictions by using more macroeconomic variables. This study can also be extended to a larger time period covering the major crises experienced in 1990s to see the prediction capabilities of different methods such as VAR, ANN, or other alternative methods. By extending this study with more variables and more countries covering a larger time interval, a potential global crisis can even be predicted prior to its occurence. Consequently, long-term forecasting will contribute positively to the global economy as a first step of providing stability which is needed in the international business and economic environment.

## Figures and Tables

**Table 1:** Results for stable and unstable situations based on simulations for the chosen VAR(2) model.

	Lag length					
Information criterion	0	1	2	3	4	5
Frequency distribution of estimated VAR orders, *T* = 180						
HJC, stable VAR	3.25	4.0	97.5	4.5	2.0	0.3
HJC, unstable VAR	0.1	3.3	95.3	4.3	3.1	0.2

HJC signifies the Hatemi-J information criterion presented by (2).

**Table 2:** Augmented dickey fuller (ADF) stationary test results.

Variables	Level value			First difference		
	None	Intercept	Trend and intercept	None	Intercept	Trend and intercept
USD	1.0078(0)	−2.0151(0)	−2.7693(0)	−9.22638(0)^*∗*^	−9.3790(0)	−9.357046(0)
	0.9172 (*p* value)	0.2802 (*p* value)	0.2107 (*p* value)	0.000 (*p* value)	0.000 (*p* value)	0.000 (*p* value)

BIST	1.5502(0)	0.0584(0)	−3.0669(1)	−11.1903(0)^*∗*^	**−**11.3511(0)	−11.3634(0)
	0.9702 (*p* value)	0.9616 (*p* value)	0.1176 (*p* value)	0.000 (*p* value)	0.000 (*p* value)	0.000 (*p* value)

GP	1.3376(1)	0.3920(1)	−2.0456(1)	−9.9681(0)^*∗*^	−10.2438(0)	−10.2221(0)
	0.9543	0.9066 (*p* value)	0.5719	0.000	0.000	0.000

“*∗*” means meaningful according to 5% significance level.

**Table 3:** The results of variance decomposition.

Period	S.E.	DGOLDPRICE	DBIST	DUSD
Variance decomposition of DGOLDPRICE				
1	2.326149	100.0000	0.000000	0.000000
2	2.418729	99.52846	0.337312	0.134227
3	2.426568	99.31653	0.455137	0.228337
4	2.431537	99.20369	0.481523	0.314782
5	2.431664	99.20029	0.481729	0.317978
6	2.431714	99.19671	0.483779	0.319516
7	2.431728	99.19564	0.484172	0.320188
8	2.431729	99.19562	0.484173	0.320207
9	2.431729	99.19561	0.484181	0.320211
10	2.431729	99.19560	0.484183	0.320213

Variance decomposition of DBIST				
1	3907.536	14.89173	85.10827	0.000000
2	3958.518	14.65356	84.73305	0.613395
3	4007.277	16.04923	83.27992	0.670842
4	4015.353	16.37279	82.94525	0.681959
5	4015.574	16.37697	82.93850	0.684534
6	4015.730	16.37801	82.93413	0.687867
7	4015.753	16.37849	82.93319	0.688321
8	4015.755	16.37852	82.93316	0.688321
9	4015.756	16.37852	82.93315	0.688337
10	4015.756	16.37852	82.93314	0.688340

Variance decomposition of DUSD				
1	0.055513	15.78019	14.56730	69.65252
2	0.059682	17.94952	15.26003	66.79045
3	0.059814	18.17990	15.27787	66.54223
4	0.060009	18.59504	15.27692	66.12804
5	0.060021	18.59939	15.29406	66.10655
6	0.060026	18.60796	15.29353	66.09851
7	0.060027	18.61097	15.29291	66.09612
8	0.060027	18.61101	15.29296	66.09603
9	0.060027	18.61101	15.29298	66.09601
10	0.060027	18.61102	15.29298	66.09600

Cholesky ordering: DGOLDPRICE DBIST DUSD				
